# Editorial: Understanding the impact of lung ventilation heterogeneity

**DOI:** 10.3389/fmedt.2024.1332958

**Published:** 2024-03-04

**Authors:** Brooks Kuhn, Igor Barjaktarevic

**Affiliations:** ^1^ Division of Pulmonary and Critical Care Medicine, UC Davis School of Medicine, Sacramento, CA, United States; ^2^ Division of Pulmonary and Critical Care Medicine, UCLA Geffen School of Medicine, Sacramento, CA, United States

**Keywords:** ventilation heterogeneity, COPD, asthma, mechanical ventilation, CT imaging, endotracheal tube

**Editorial on the Research Topic** Understanding the impact of lung ventilation heterogeneity

Ventilation heterogeneity (VH) is an inherent characteristic of respiratory physiology. Gravity, anatomic differences in the airway length, size and branching, differences in the size and anatomy of left and right lungs, local transpulmonary pressure gradients, and mechanical interaction with the chest wall all can lead to heterogeneous distribution of ventilation ($\dot{V}$) and perfusion ($\dot{Q}$). In conditions that lead to a significant $\dot{V}/\dot{Q}$ mismatching, the respiratory system attempts to optimize gas exchange using multiple systemic (i.e., breathing patterns and neuro-respiratory reflexes, cardiac output changes, or autonomic nervous system engagement) and local (hypoxic pulmonary vasoconstriction and hypocapnic bronchoconstriction) compensatory mechanisms. When these mechanisms are insufficient to optimize regional gas exchange, VH may become clinically relevant and contribute to the disease burden.

VH can be a consequence of *conduction*, *diffusion,* or *conduction-diffusion* processes (1): systemic and regional diseases characterized by isolated or combined diseases of the airways, lung parenchyma or pulmonary vasculature. Traditionally, the data about VH focus on airway diseases where gas maldistribution and delayed regional gas emptying lead to clinically relevant respiratory pathophysiology (2). Airway hyperresponsiveness leads to small airway obstruction from bronchospasm, and mucous plugging leads to VH in asthma (3), with VH itself leading to poor asthma control (4, 5). Cystic fibrosis and COPD (6) are marked by VH, which is a sensitive biomarker for detecting early disease (7).

More broadly, if the problem of VH is not exclusively looked at as a *problem of gas distribution* but rather as a problem of *inadequate regional gas exchange* resulting from regional mismatch between the ventilation and perfusion where compensatory mechanisms (3) fail to prevent clinically relevant respiratory compromise, VH represents a prevalent phenomenon in numerous respiratory pathologies, including both acute and chronic processes. While the distribution of lung infiltrates in acute pneumonia grossly varies and is rarely symmetric, more homogenous processes such as acute respiratory distress syndrome (ARDS), can also characterized by both diffuse and focal distribution of lung abnormalities (8). Emphysema, bronchiectasis, interstitial lung diseases, lung cancer, or pulmonary embolism are all characterized by significant distribution heterogeneity which can result in clinically significant $\dot{V}/\dot{Q}$ mismatch. While in most cases compensatory mechanisms and systemic therapeutic interventions are sufficient to attenuate the detrimental effect of hypoxemia and offer a bridge to recovery, there are situations when not adjusting the management to account for heterogeneity of pathologic processes may compromise the outcomes.

**Figure 1:**
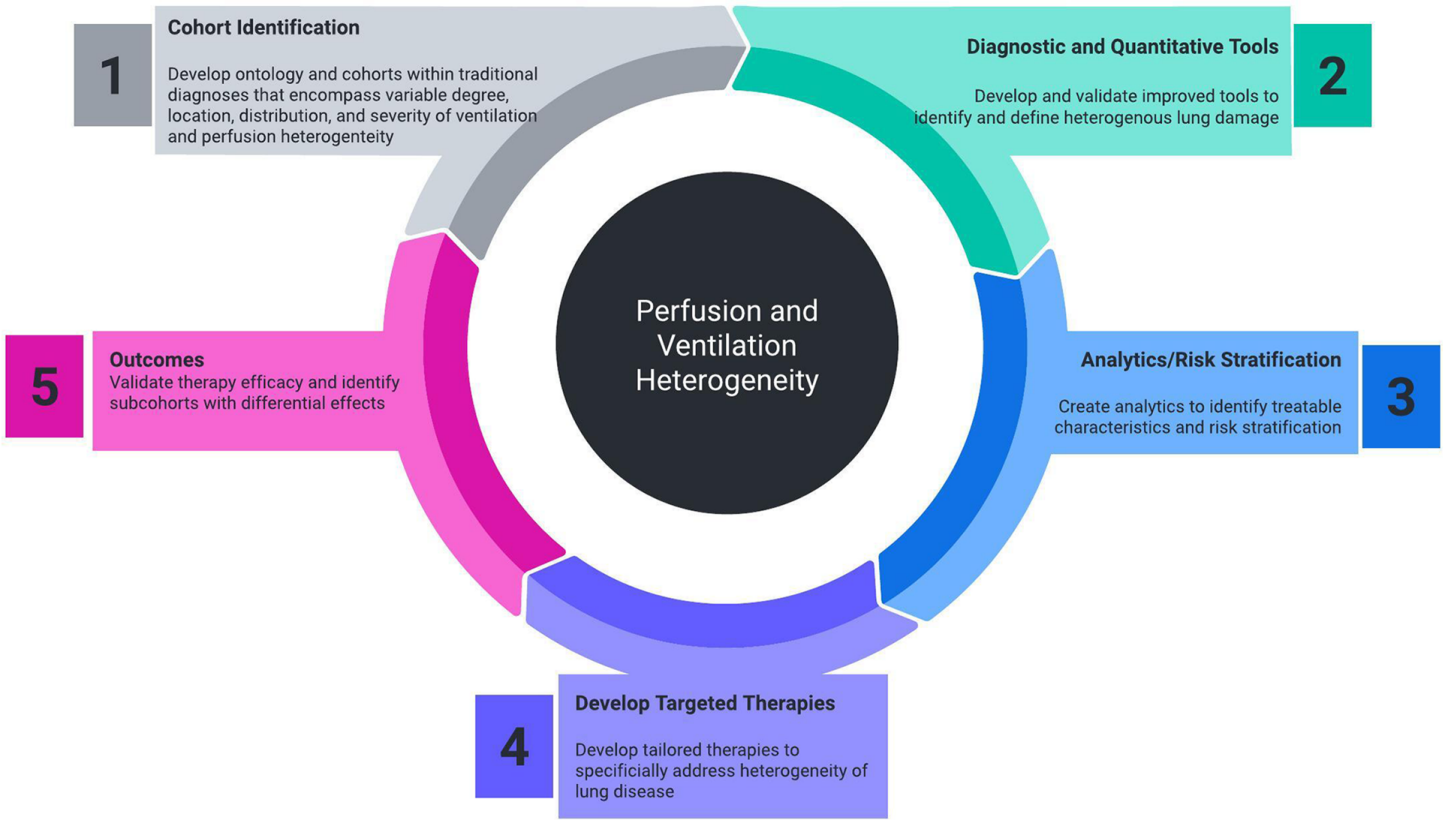
Necessary components of cohort identification, diagnostic tools, analytics, targeted therapeutics, and outcome measurement that will improve the ability to measure and treat perfusion and ventilation heterogeneity.

One of the downsides of traditional management of lung disease is the assumption that the respiratory system behaves as a single homogeneous unit. Universally treating the lungs as a single compartment needs to be challenged. The clearest example of current therapy addressing regional differences in the lung is endobronchial lung volume reduction in emphysema. Positive pressure ventilation may carry the risk of overdistension of compliant areas of the lungs in the effort to ventilate poorly compliant regions, leading to worsened $\dot{V}/\dot{Q}$ mismatch and ventilator-induced lung injury (9). Despite the prevalence, there exists a need for improved diagnostic, quantitative, and therapeutic technologies to better study and treat VH (Figure 1).

In this Research Topic of *Frontiers in Medical Technology*, six groups share their efforts to develop methods and tools to better detect, quantify, and treat the impact of heterogenous lung disease. Lauria et al. discuss the approach of measuring tissue elasticity—a biomechanical property measured from 5DCT CT imaging—to assess interlobar and intralobar heterogeneity**.** Evaluating patients with COPD, a significant variability in elasticity throughout regions in the lung was found with more pronounced variability in regions with more severe emphysema. The ability to reliably measure this heterogeneity may offer actionable information during surgical planning for lung sparing resection of tumors. Ishrak et al. used doppler radar remote sensing of torso kinematics to indirectly measure cardiopulmonary function by tracking body surface motion due to heart and lung activity. Doppler radar may offer a non-invasive, contactless, and flexible method to respiratory monitoring and may represent a promising tool with applications to remote patient monitoring and self-management. Karmali et al. summarized the state of various dynamic imaging modalities with the potential to assess regional lung ventilation and physiology: xenon 133-computed tomography, 4D computed tomography, hyperpolarized (helium-3 and xenon-129) gas magnetic resonance imaging, and x-ray velocitrometry. The review highlights diseases such as COPD that manifest with non-uniform ventilation and perfusion and require the measurement and identification of regional deficits that cannot be detected by spirometry or static CT scans. Novel imaging diagnostic approaches (radioisotope enhanced functional MRI or CT imaging, electrical impedance tomography) add the ability to better understand spatial heterogeneity of ventilation and add to the information about temporal VH that traditional diagnostic tests such as inert gas washout techniques provide. Kirkness et al. compared a novel approach to measuring regional lung ventilation via x-ray velocimetry ventilation (XV) analysis to spirometry. XV analysis, which measures lung tissue motion during breathing, offers high spatial resolution and does not require contrast agents, breath-hold maneuvers, and has a low dose of radiation required. While further research and validation are needed across a variety of lung conditions, this study shows the potential of XV analysis as a sensitive and efficient tool for monitoring lung health in patients with heterogenous lung disease, with potential diagnostic and research applications. Nwajuaku et al. discuss the development and benefits of the sOLVe (simple One Lung Ventilation for everyone) endotracheal tube, a novel dual-lumen tube potentially applicable in broader clinical settings including non-surgical candidates. The sOLVe tube offers key innovations, including a single size tube that fits an array of patient sizes, a more flexible silicone shaft, enhanced stability in the airway through trapezoidal balloon cuffs to prevent dislodgement, and a unique lumen shape to allow catheters and larger bronchoscopes, the FDA deems the device substantially equivalent, but further studies are planned to evaluate its performance compared to tradition dual lumen tubes in clinical settings. System for asymmetric flow regulation (SAFR) aims to address the problem of uneven distribution of air in the lungs of mechanically ventilated patients with asymmetric distribution of acute lung injury (Barjaktarevic et al.). SAFR, in combination with dual lumen endobronchial tubes, is proposed as a method to ventilate the left and right lungs independently and uniquely. In this study, preclinical experiments were conducted to assess SAFR's effectiveness in redistributing air between simulated lungs. The impacts of this capability are broad and significant, offering the ability to bring precision treatment to mechanical ventilation.

In conclusion, the assessment of lung tissue heterogeneity in diagnostic and therapeutic approaches holds promise. Understanding and accounting for regional variations in lung physiology, as determined by factors like tissue elasticity, can lead to more precise and effective diagnostic and treatment strategies, ultimately leading to more personalized care. The six contributions in this Research Topic highlight the exciting developments in medical technologies promising to bring this promise to reality.
